# Challenges in the Understanding of Oligometastatic Disease in Clinical Practice

**DOI:** 10.7759/cureus.72500

**Published:** 2024-10-27

**Authors:** Inmaculada Navarro-Domenech, Aisling Barry, Jane Tsai, Grace Ma, Philip Wong

**Affiliations:** 1 Radiation Oncology, Hospital Universitario La Paz, Madrid, ESP; 2 Department of Radiation Oncology, Princess Margaret Cancer Centre, Toronto, CAN; 3 Department of Radiation Oncology, Cork University College, Cork, IRL; 4 Radiation Medicine Program, Princess Margaret Cancer Centre, Toronto, CAN; 5 Department of Family and Community Medicine, Freeman Centre for the Advancement of Palliative Care, North York, CAN

**Keywords:** clinical practice, local therapy, oligometastatic disease, quality improvement study, survey.

## Abstract

Introduction*:* There is little data describing oligometastatic disease (OMD) and decision-making. We sought to understand the knowledge gaps and challenges in deciphering and delivering treatments.

Materials and methods: This is a quality improvement (QI) study conducted via an anonymous survey. Three different clinical scenarios of OMD (oligo-recurrence disease, synchronous de-novo OMD, and oligo-progressive disease) were presented to assess participants’ comprehension. A qualitative approach was used, involving four open-ended questions. Summary statistics and descriptive analysis were utilized to describe survey answers.

Results: The survey was answered by 70 clinicians, 56% (n=39) medical oncologists, 24% (n=17) radiation oncologists, 7% (n=5) surgeons, and the remaining 13% (n=9) from anatomical pathology, radiology, and palliative care. The three clinical cases were correctly identified as oligo-recurrence, de-novo, and oligo-progression disease in 63% (n=44), 94% (n=66), and 76% (n=53) of responses, respectively. Additionally, for each case, the majority of respondents indicated that they would offer local treatment (n=59, 84%; n=57, 81%; n=55, 79%, respectively). Seventy-nine percent (n=49) perceived differences between each modality of local therapies. Physicians perceived challenges including the lack of prospective trial data and unclear approach to OMD. An important determinant in deciding whether patients may benefit from treatment was tumor histology.

Conclusion: The term OMD involves certain difficulties in definition and management. Positive and negative trials have further added uncertainty regarding who would best benefit from local treatment. The discordance in outcome expectations from physicians and patients will need to be addressed to ensure that patient’s goals of care are met.

## Introduction

The term oligometastatic disease (OMD) describes a state between localized and metastatic disease [1]. Recently, the European Society for Radiotherapy and Oncology and the European Organisation for Research and Treatment of Cancer (ESTRO/EORTC) groups described subgroups within the OMD term, based on the timing of the development of metastatic disease [2].

Metastasis-directed therapy (MDT) involves local therapy to oligometastatic lesions, including surgery, stereotactic body radiation therapy (SBRT), radiofrequency ablation (RFA), etc. Disease control, consolidative therapy in patients with partial responses to systemic treatments, symptom management, and oncological emergencies are the main indications for MDT. However, in some cases, it is proposed as a “curative/ablative” treatment for OMD [3,4]. There is notable evidence suggesting that MDT can positively affect patient survival [5,6]. Despite the increasing number of clinical publications in this field, local radical therapy is often recommended for OMD in clinical guidelines, at least in some solid tumors such as non-small cell lung cancer (NSCLC) or prostate cancer [7,8]. Some data support the use of MDT in the multidisciplinary application of oligometastatic treatment and decision-making [9]. There are concerns about toxicity, and the main recommendation is to administer it within clinical trials. However, not all patients are eligible for enrollment in clinical trials, and many are treated off-study, guided by previous study outcomes [6-8], though this approach may introduce some potential for clinical bias.

The aim of this project was to evaluate the current clinical practice in OMD among different specialties involved in managing and delivering treatments to patients with oligometastatic cancer. We also aimed to explore the expectations and challenges that physicians perceive in their practice in this specific oncological entity.

This article was previously presented as a meeting abstract under the title “Real World Challenges in Defining Oligometastatic Disease in Clinical Practice” at the 2023 ASCO Annual Meeting on May 31, 2023; and under the title of “Challenges in Defining Oligometastatic Disease in Clinical Practice” at 2023 CARO-COMP Joint Scientific Meeting on September 21, 2023.

## Materials and methods

This is a single institution quality improvement (QI) study conducted via an anonymous electronic survey (SurveyMonkey®). The study was reviewed and approved by the University Health Network (UHN) Quality Improvement Review Committee (QIRC) (ID# QIRC 22-0421).

The UHN is the largest academic health science center in Canada, based in Toronto. UHN is a conglomerate of multiple hospitals (Toronto General Hospital, Toronto Western Hospital, Princess Margaret Cancer Centre, and Toronto Rehabilitation Institute) and research institutes, providing both tertiary and quaternary care, along with world-leading research and education programs.

The survey was emailed in October 2022 to UHN multidisciplinary staff in Toronto, which included: Medical Oncology, Radiation Oncology, Surgical Oncology, Palliative Care, Radiology, and Pathology. Participation was optional.

The design of the study was a structured survey with both multiple-choice and open-ended questions. It consisted of three anonymous OMD clinical cases, with three multiple-choice questions and five open-ended questions, including an optional feedback comment/suggestion (Appendix 1). It was estimated to take between 10 and 20 minutes to complete. Participants were asked to reflect on professional experiences related to the challenges associated with the management of OMD. The questions were based on the definition and the potential role of local treatments for OMD.

An introductory email described the survey background. Physician consent was obtained prior to access and completion of the survey. The survey was emailed to staff and fellows, of the UHN team.

Descriptive summary statistics were used for categorical variables using counts and percentages. In order to simplify the answers to the open-ended questions, these were reviewed, grouped together based on category, and analyzed using sector and bar diagrams, the decision-tree method, and sorted by prevalence; grouped in descending order, with those with the highest matching responses coming first. Two independent readers with qualitative research experience (JT, GM) further revised the categorization of open answers.

## Results

 A total of 70 surveys were completed (response rate 23%), with 39 surveys (55.71%) from Medical Oncology, 17 (24.29%) from Radiation Oncology, 5 (7.14%) from Cancer Surgery, 4 (5.71%) from Cancer Anatomical Pathology, 3 (4.29%) from Radiology, and 2 (2.86%) from Palliative Medicine.

Clinical cases

The first clinical case described repeat oligo-recurrence disease, based on the ESTRO/EORTC classification. Forty-four (62.86%) participants correctly identified the classification of the case.

Local treatment as a radical treatment or treatment prior to a new line of systemic treatment was considered by 59 (84.29%) participants. Reasons stated for considering local treatment were for local control (27/70 (38.57%)) and 22 physicians (31.43%) described advantages in terms of local control, survival, and quality of life (QoL).

The second case reported synchronous de-novo OMD. The majority of participants agreed with this diagnosis (66/70 (94.29%)). Fifty-seven (81.43%) participants considered local therapy as the primary management option for the primary tumor and the oligometastatic lesion. A third (N = 22, 31.43%) of participants described their outcome reason as local control only and a third (N = 22, 31.43%) described an advantage in survival and QoL, too.

Finally, the third case described oligo-progression disease. Fifty-three (75.71%) participants identified this correctly. Of those who answered, 55 (78.57%) stated that local treatment was the most appropriate treatment option in addition to systemic treatment. Nearly half of the participants (48.57%) described local control as being the primary outcome. All response data are summarized in Table 1.

**Table 1:** Response data from clinical cases

Question	Answers	Response Rate
Clinical Case A	De-novo oligometastatic disease	5.71% (n=4)
	Induced oligometastatic disease	1.43% (n=1)
	Oligo-recurrence disease	62.86% (n=44)
	Oligo-progression disease	30% (n=21)
Would you consider local treatment for case A?	Yes	84.29% (n=59)
	No	15.71% (n=11)
What are the reasons why are you considering a local therapy in case A?	I think there is a local control advantage	38.57% (n=27)
	I think there is a survival advantage	5.71% (n=4)
	I think there is a quality-of-life advantage	10% (n=7)
	All above	31.43% (n=22)
	None of them	14.29% (n=10)
Clinical Case B	De-novo oligometastatic disease	94.29% (n=66)
	Induced oligometastatic disease	2.86% (n=2)
	Oligo-recurrence disease	2.86% (n=2)
	Oligo-progression disease	0% (n=0)
Would you consider local treatment for case B?	Yes	81.43% (n=57)
	No	18.57% (n=13)
What are the reasons why are you considering a local therapy in case B?	I think there is a local control advantage	31.43% (n=22)
	I think there is a survival advantage	7.14% (n=5)
	I think there is a quality-of-life advantage	11.43% (n=8)
	All above	31.43% (n=22)
	None of them	18.57% (n=13)
Clinical Case C	De-novo oligometastatic disease	4.29% (n=3)
	Induced oligometastatic disease	10% (n=7)
	Oligo-recurrence disease	10% (n=7)
	Oligo-progression disease	75.71% (n=53)
Would you consider local treatment for case C?	Yes	78.57% (n=55)
	No	21.43% (n=15)
What are the reasons why are you considering a local therapy in case C?	I think there is a local control advantage	48.57% (n=34)
	I think there is a survival advantage	10% (n=7)
	I think there is a quality-of-life advantage	5.71% (n=4)
	All above	15.71% (n=11)
	None of them	20% (n=14)

Open-ended questions

The four open-ended questions and the response rate are depicted in Table 2.

**Table 2:** Statement and response rate of the clinical cases and the four open-ended questions. QoL: quality of life; SBRT: stereotactic body radiotherapy; RFA: radiofrequency ablation.

Question	Statement	Response rate
Clinical Cases	Please answer the questions based on the clinical cases A, B, and C: Which of the previous clinical cases describes better the following oligometastatic categories? Would you consider local treatment (e.g. surgery, SBRT, interventional radiology procedures) for the metastatic lesions? What are the reasons why are you considering a local therapy for the previous metastases?	70/70 (100%)
Q1	Do you perceive differences (e.g., survival, local control, QoL) between local therapy options (e.g., surgery versus SBRT versus RFA)? Which would you consider is the main advantage of each one?	62/70 (89%)
Q2	In the setting of metastatic disease, what is the maximum number of metastases you consider to define oligometastatic disease? Please, type a number from 0 to 10	67/70 (96%)
Q3	What are the challenges you experience in your practice when managing oligometastatic patients?	63/70 (90%)
Q4	What do you consider is the most important variable in determining which oligometastatic patient would benefit the most from local treatments of metastases (e.g., any patient characteristics, tumor characteristics, molecular mutations)? Are there histologies in which you expect a greater benefit from SBRT for oligometastatic diseases?	63/70 (90%)
Q5	We sincerely thank you for your participation in this study. Your feedback is very valuable to us. Please provide any additional comments or suggestions below (optional).	19/70 (27%)

**Figure 1:**
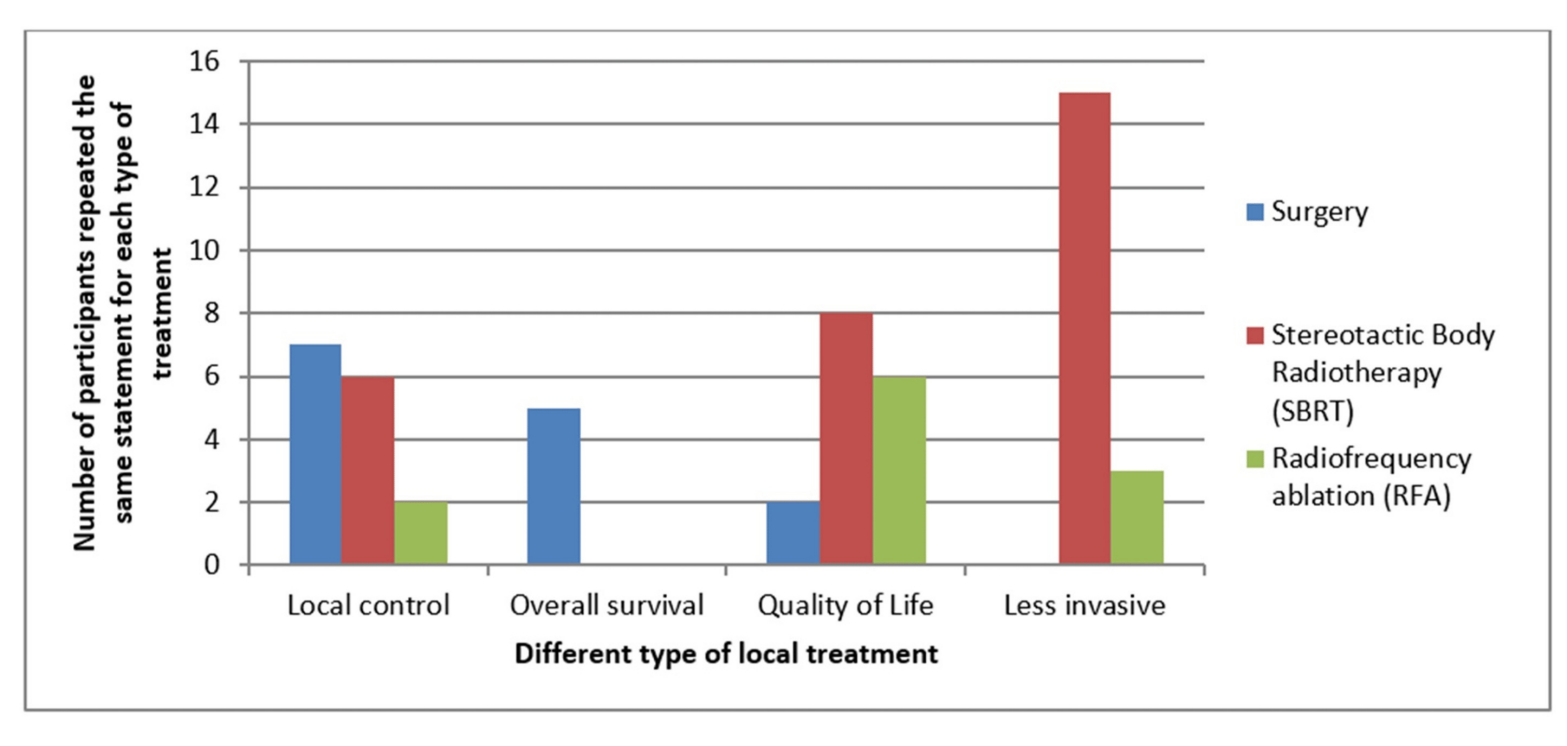
A bar diagram illustrating the main advantages of different local treatments (surgery, SBRT, and RFA) is presented. Each bar represents the frequency at which participants repeated the same statement for each type of local treatment. SRBT: Stereotactic body radiotherapy; RFA: Radiofrequency ablation

Question 1 was answered by 89% (N=62) of participants, of which forty-nine (79%) perceived differences in outcomes between local therapies. The main differences between the local treatments (surgery, SBRT, and RFA) were local control (LC), overall survival (OS), quality of life (QoL), and invasion/toxicity (Figure 1).

**Figure 2:**
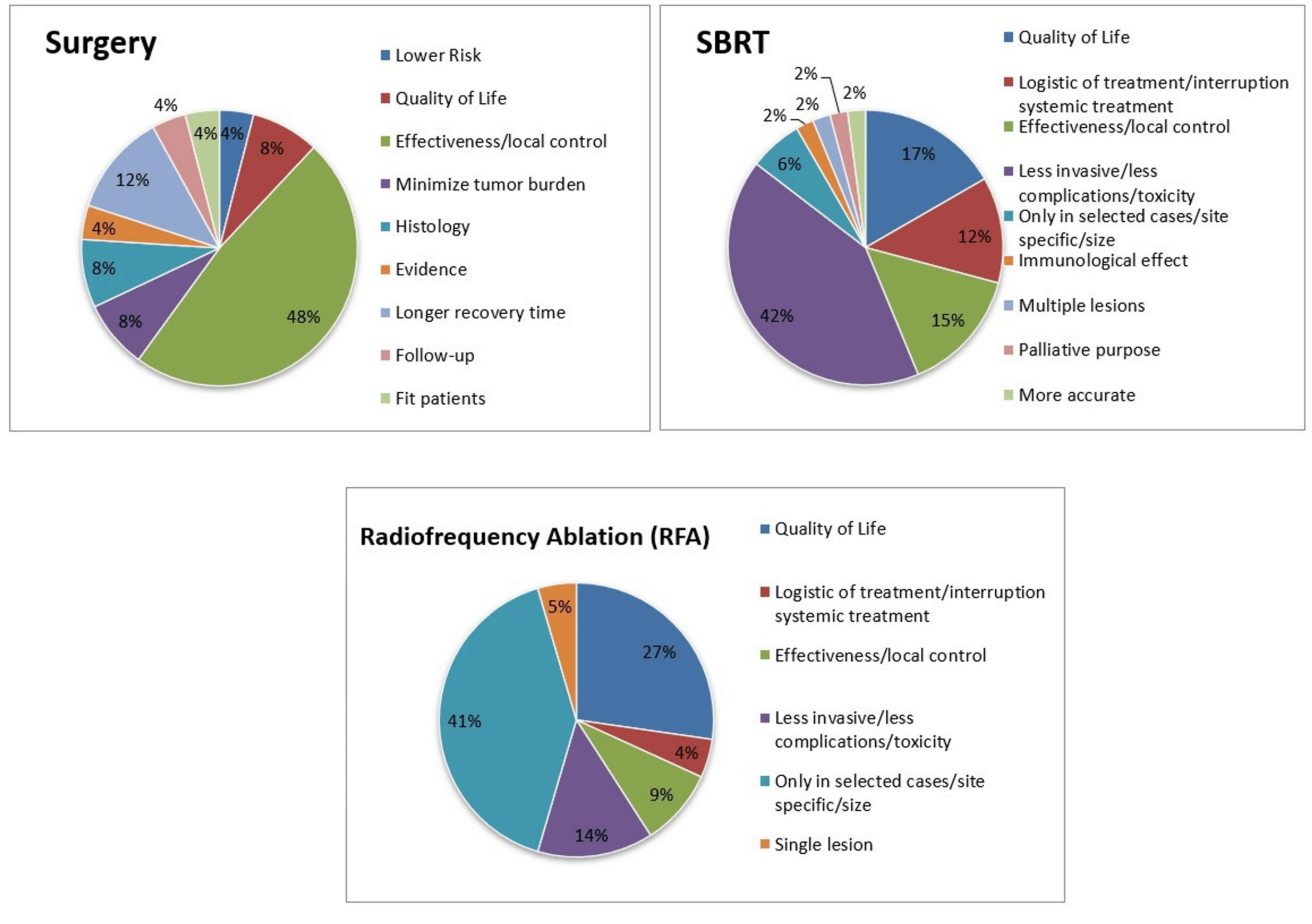
A pie chart that collects all the responses stratified by each type of local treatment.

All the answers stratified by each type of local treatment are depicted in Figure 2.

**Figure 3:**
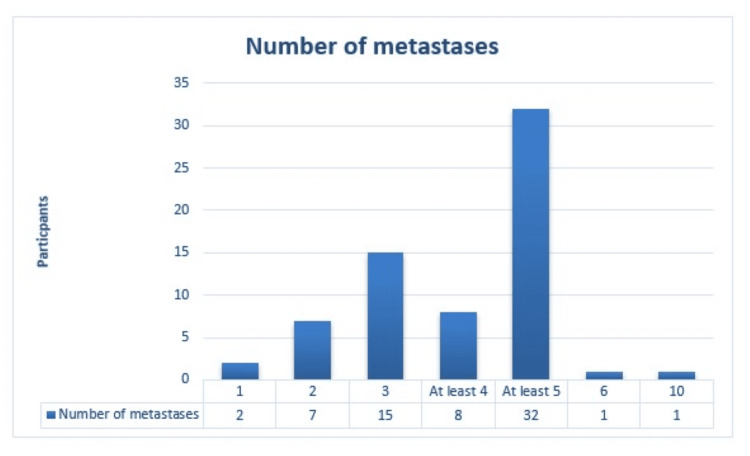
Bar chart showing the number of metastases considered to define oligometastatic disease (OMD).

In question 2, the majority (95.5%, n=64/67 responses) considered at least five metastases as defining OMD (Figure 3). 

Question 3 was answered by 63/70 participants (90%). Challenges were grouped according to time during the treatment setting: pre-treatment, during treatment, and post-treatment (Table 3).

**Table 3:** Challenges in the management of OMD according to pre-treatment, during treatment or post-treatment settings, including some examples of answers. OMD: Oligometastatic disease; MDT: Metastasis-directed therapy; PFS: Progression-free survival; OS: Overall survival; SBRT: Stereotactic body radiotherapy; RT: Radiation treatment

Treatment Phase	Theme Identified	Challenge Defined
Pre-treatment (Treatment decision-making/referring the patient)	Lack of scientific evidence	Unclear definition and classification of OMD, e.g., definition may be somewhat more fluid than the literature suggests, agreement of the definition of oligometastatic between specialists within the MDT.
		Lack of clinical and prospective trial data, e.g., the studies are small, and prospective data are heterogeneous. Therefore, it is difficult to know if there is a survival advantage to the approach and how to select patients who will get the maximum benefit out of it.
		Variable biases and policies in patient selection, e.g., the data is sparse, and patient factors (fitness for intervention) can preclude you from aggressive treatment.
	Unclear treatment objectives	Switching systemic therapy instead of offering local control, e.g., to convince my medical oncologist colleagues that we should attempt to offer local control, versus switching systemic therapy.
		Negative perception/unclear goals of local treatment, therefore lack of referrals: No correlation with PFS/OS. Aggressive local treatment, treatment of asymptomatic lesions, ablation vs consolidation, risk of over-treating, e.g., it has frequently become a response to an imaging finding that pays little attention to the understood natural history of the disease. There is a great deal of indication creep taking place. Does not correlate to PFS/OS advantage, and usually means have to change systemic therapy as well. A good option for locoregional control and/or symptom control, oligometastases are NOT hard to manage. Oligo-progression is a mess, no one knows who benefits, etc.
	Lack of prognostic guidance	Related to the biology, the prognosis of the tumor and chronology of metastatic disease, e.g., understanding the biology and differentiating aggressive vs indolent oligometastases; outcomes not correlating with imaging necessarily - need better prognostication to decide whom to give systemic treatment to alone versus adding local treatment; distinguish cases that will soon have extensive progression.
	Disagreement of decision	Multidisciplinary opinions, e.g., I think mainly convincing surgeons to operate.
During treatment (Clinical management)	Technique specialization	This includes different areas of treatment (e.g., spine, gastrointestinal, etc.) and expertise, e.g., multi-site/multi-organ location of oligometastases that qualify for SBRT. Different SBRT considerations based on location (sim, dosimetry, targeting, etc.). An unwillingness to try oligometastatic therapy outside of the trial setting.
		Different facilities have differing resources and capabilities, accessibility to techniques, e.g., different practices in different institutions, and different physicians within facilities.
	Logistic	Patient and diagnosis-related bias, e.g., to ensure we did not miss other sites of metastases; the site of oligometastases may be within a prior radiation field.
		Lack of a standardized protocol: Different management, different institutions, e.g., each case is unique, lack of good guidelines, very institution dependent.
	Management during the treatment course	Increase the toxicity, e.g., with the improvement of systemic treatment, its contribution may be reduced and toxicity may increase.
		Interruption of systemic treatment and/or delays in the treatment, e.g., the main challenge is to not delay systemic therapy too much (i.e., an effective targeted therapy) to offer local control if no local symptoms. In some cases, e.g., resecting liver metastases, it requires extensive collaboration between different specialties in order to coordinate the timing of chemotherapy and surgery; delays in proceeding with surgery/RT for oligometastatic disease can be difficult for patients.
Post-treatment (Clinical disease outcomes and follow-up)	Lack of response and side effects	Patients and physicians’ expectations in survival outcomes, e.g., patients may have a poor understanding of their disease and it can be difficult to manage expectations with these treatments.
		Cases that will soon have extensive progression, e.g., long-term control and development of new lesions.
	Management after local treatment	Continue on systemic treatments vs observation, e.g., knowing when to switch systemic therapy.

Question 4 was answered by 63 (90%) participants. The most important variable and histologies in determining which oligometastatic patient would benefit the most from local treatments were analyzed in Table 4.

**Table 4:** Variables that determine the benefit of OMD treatment according to participants’ common themes’ answers. OMD: Oligometastatic disease

Ranking of the most repeated answers	Variables determining the benefit of oligometastases (OM) treatment based on common themes from participants’ responses	Number of participants
1	Tumor histology and molecular profile/positive hormone receptor	31
2	Tumor biology (tumor kinetics growth, indolent vs aggressive) and radiosensitivity	23
3	Patient’s characteristics (performance status, age and quality of life, if the patient is on systemic or targeted molecular treatment, goals of care, symptoms, prior radiotherapy to the area, comorbidities for surgery)	20
4	Oligometastases characteristics (OM classification, number, size, pattern, disease burden and location)	13
5	Primary tumor control and interval time to progression	12
6	Response and options of systemic treatment (targeted therapy and immunotherapy). Evidence data for survival benefit (colorectal cancer and lung cancer vs. breast cancer)	9

Participants described a number of different histologies including breast, prostate, kidney, melanoma, squamous cell carcinoma, testicular cancer, Merkel Cell carcinoma, NSCLC, and lymphomas. However, no single predominant histology was identified as expected to derive a greater benefit from local treatment.

## Discussion

This survey aims to assess the current understanding of OMD among medical professionals specialized in cancer patient care. Despite the publication of various classifications [2,10] and studies on local treatments for OMD [6,11,12], our analysis of this survey reveals discordance in OMD definition and clinical procedures for managing patients with OMD.

Based on the obtained answers, de-novo OMD and oligo-progression disease are easier to diagnose, while oligo-recurrence disease is more challenging. Additionally, the majority agreed that treating these lesions can result in better local control. However, this may differ depending on the classification of OMD and prognosis [13,14]. The concept of oligo-progressive disease and its benefit from MDT is unclear [15]. However, the CURB trial randomized oligo-progressive breast and NSCLC to receive standard of care (SOC) treatments vs. SOC plus SBRT. While NSCLC patients demonstrated a significantly superior progression-free survival (PFS) (10 vs 2.2 months; p=0·0039), no significant difference was found in breast cancer (4.4 vs. 4.2 months; p=0.43) [16]. In light of this, not only is the characterization of OMD crucial in determining outcomes, but histology may also play an important role. In addition, the number, size, and affected organ may influence the MDT response [17,18]. There is a discrepancy in the number of metastases that define OMD. Among the various definitions, it is considered a limited number of metastases (a small number of low-volume metastases, at least 3 or 5), a single or limited number of sites/regions/organs, or a limited number of distant metastatic regions (typically ≤5) that contain the primary tumor [13]. The ESTRO-ASTRO consensus collected all these possibilities and reported that the definition of OMD is independent of primary tumor type, histology, or the metastatic site(s); and that the feasibility of delivering radical treatments determines the maximum number of lesions and sites that can be treated [10]. Currently, the impact of SBRT on up to 10 lesions is being studied [19]. This is also important because the response of the OMD to a local treatment is considered as a prognostic factor [17].

When analyzing the differences between the different MDT options according to the participants, surgery appears to be considered more effective in local control and OS, while SBRT is perceived as the least invasive and leads to higher QoL. RFA is only considered in specific cases such as liver metastases, which are also associated with good QoL but lower disease control. Although there are no randomized controlled trials that compared disease control between surgery and SBRT, some publications have reported outcomes in the use of SBRT for OMD that demonstrate efficacy in local control and even OS, comparable to the surgery [2,14,19-21].

Due to the difficulties observed in clinical practice in the management of OMD, we conducted this survey to analyze the factors that professionals consider most challenging. The majority agreed that there is a lack of scientific evidence and an unclear definition of OMD, which makes it difficult to select patients and define treatment objectives. Additionally, the approach may vary depending on the center where the treatment is being conducted. The challenges reported in the practice of managing OMD patients in our survey are similar to those reported in the literature [20]. These include accurate staging and diagnosis, treatment selection, balancing local and systemic therapies, monitoring and follow-up, psychosocial and emotional aspects, and shared decision-making. These challenges highlight the importance of a multidisciplinary approach to OMD management, as it involves different medical specialties and aspects of patient care.

Regarding the variables that are considered important in determining the benefit of local treatments for OMD, the answers obtained align with the current evidence [19,22]. Tumor characteristics, molecular mutation, patient characteristics, treatment goals, and multidisciplinary assessment are all important factors that should be taken into consideration. The lack of consensus on the indication and selection of localized treatment, and its benefits, reflects the complexity of OMD management, and the need for personalized treatment plans that take into account individual patient characteristics and goals of care.

Overall, this survey highlights the need for continued education and discussion among medical professionals regarding OMD management. The discrepancies observed in the understanding and clinical procedures of OMD indicate the need for more standardized guidelines, prospective clinical trials and multidisciplinary collaboration to provide the best possible care for patients with this complex disease state [23].

In addition, in light of these concerns, a recently published survey gathered perspectives from 44 oncologists on the curability and treatment decision-making for the different subtypes of OMD [24]. The perception is that oligo-progressive disease has a poorer response compared to other subtypes, which has also been reported in some publications [25], and is under study in numerous prospective trials (e.g., NCT04122469). However, depending on the histology, an improvement in PFS has been described with the treatment of oligo-progression using SBRT [16]. Nonetheless, the role as “ablative” or “consolidative” local treatment associated with immunotherapy is not well-stablished [26]. Given the published data, a multidisciplinary approach should be taken for the decision-making regarding local treatment integrated with systemic treatment. Health-related quality-of-life (HRQOL) was assessed between responders and non-responders to SBRT for OMD, showing no clinically significant deterioration [27].

One of the limitations of this study is the low response rate [28,29]. This may be related to the limited time available for healthcare professionals and, that not all physicians who received the survey were involved in managing OMD. Furthermore, this study was based on the opinions of professionals from a single health system which may bias that results and limit the generalizability of the conclusions. However, it is the largest survey for OMD conducted to date [24]. Furthermore, the diversity of disciplines can restrict the interpretation of the results, as each discipline reports its own experiences. Nevertheless, the collective opinion reflects the varied understandings and expectations from oncologists who care for OMD patients within a single cancer center.

There are key questions in OMD that remains unclear. In our research, we have identified several critical knowledge gaps that warrant further investigation and collaboration within the medical community in order to be addressed. First, we need to explore the impact of SBRT on local control, OS and QoL, across the different cancer histologies and in comparison, to other local treatments such as surgery. This assessment is essential to understand whether SBRT offers a balance between reducing LC and OS while enhancing QoL. Second, the definition of OMD as 1-5 metastatic lesions across all cancer histologies requires careful consideration. It is essential to determine if this definition adequately represents OMD for various cancer types, at the site of metastasis implantation and/or in terms of the size. Additionally, we need to explore the role of tumor histology and molecular profiling in determining OMD's natural progression and response to localized treatments. This aspect is essential for identifying the most effective local therapy approaches for individual patients and developing standardized response criteria and the benefit from local treatments.

These identified knowledge gaps offer significant opportunities for hypothesis exploration and collaborative researches. Addressing these gaps collectively will enhance our understanding of OMD and its management within the medical community.

## Conclusions

Defining and managing OMD is challenging. It seems that mixed trial results may have increased uncertainty about which patients would benefit most from local treatments.

In this quality-improvement survey, we found a significant discordance in outcome expectations among physicians, that will need to be addressed to ensure that patients’ goals of care are met.

## Appendices

Appendix 1: Questionnaire

1. Please, which is your medical specialty? Choose your specialty.

- Medical oncology

- Cancer surgery

- Palliative medicine

- Radiology

- Cancer anatomical pathology

- Radiation oncology

2. Please, read the following clinical cases and answer the questions:

Case A: A 57-year-old woman with initial diagnosis of metastatic colorectal cancer with metastases to the liver. She completed neoadjuvant chemotherapy, followed by metastasectomy to the liver metastases and resection to the primary colorectal cancer. After completing treatment, she was off systemic treatment and five months later, she developed progression to an aortocaval lymph node metastasis.

a. Which of the previous clinical cases describe better the following oligometastatic categories?

☐ De-novo oligometastatic disease

☐ Induced oligometastatic disease

☐ Oligo-recurrence disease

☐ Oligo-progression disease

b. Would you consider local treatment (e.g., surgery, SBRT, interventional radiology procedures) for the metastatic lesions?

☐ Yes

☐ No

c. What are the reasons why are you considering a local therapy for the previous metastases?

☐ I think there is a local control advantage

☐ I think there is a survival advantage

☐ I think there is a quality-of-life advantage

☐ All above

☐ None of them

Case B: A 43-year-old premenopausal woman with a new diagnosis of metastatic multicentric cT3 N1, ER 91 to 100%, HER-2/neu negative, invasive ductal carcinoma of the right breast with a metastasis in the right proximal humerus based on whole body bone scan, X-rays and MRI.

a. Which of the previous clinical cases describe better the following oligometastatic categories?

☐ De-novo oligometastatic disease

☐ Induced oligometastatic disease

☐ Oligo-recurrence disease

☐ Oligo-progression disease

b. Would you consider local treatment (e.g., surgery, SBRT, interventional radiology procedures) for the metastatic lesions?

☐ Yes

☐ No

c. What are the reasons why are you considering a local therapy for the previous metastases?

☐ I think there is a local control advantage

☐ I think there is a survival advantage

☐ I think there is a quality-of-life advantage

☐ All above

☐ None of them

Case C: A 58-year-old man with a history of resected melanoma to the left postauricular. Ten years later, he developed progression to a conglomerate of lymphatic nodules in the retroperitoneum and left adrenal gland, biopsy proven of melanoma origin, BRAF positive V600E mutation. He started on immunotherapy. In the re-staging exam, he has mixed responses: improvement to the retroperitoneal lymph nodes, but interval increase in the left adrenal gland metastasis.

a. Which of the previous clinical cases describe better the following oligometastatic categories?

☐ De-novo oligometastatic disease

☐ Induced oligometastatic disease

☐ Oligo-recurrence disease

☐ Oligo-progression disease

b. Would you consider local treatment (e.g., surgery, SBRT, interventional radiology procedures) for the metastatic lesions?

☐ Yes

☐ No

c. What are the reasons why are you considering a local therapy for the previous metastases?

☐ I think there is a local control advantage

☐ I think there is a survival advantage

☐ I think there is a quality-of-life advantage

☐ All above

☐ None of them

3. Do you perceive differences (e.g., survival, local control, quality of life) between local therapy options (e.g., surgery versus SBRT versus RFA)? Which would you consider is the main advantage of each one?

4. In the setting of metastatic disease, what is the maximum number of metastases you consider to define oligometastatic disease? Please, type a number from 0 to 10: Click here to enter text.

5. What are the challenges you experience in your practice when managing oligometastatic patients? 

6. What do you consider is the most important variable in determining which oligometastatic patient would benefit the most from local treatments of metastases (e.g., any patient characteristics, tumor characteristics, molecular mutations)? Are there histologies in which you expect a greater benefit from SBRT for oligometastatic diseases?

We sincerely thank you for your participation in this study. Your feedback is very valuable for us. Please provide any additional comments or suggestions below (optional).
